# Volunteers, religious communities and users representatives as an alternative for visiting hospitalized patients: The importance of an infection control training

**DOI:** 10.1371/journal.pone.0286002

**Published:** 2023-05-22

**Authors:** Audrey Maurand, Ronan Le Guen, Celine Sakr, Nadine Sabourin, Bruno Hacquin, Stéphanie Boulmier, Christèle Bonnin, Lamnakhone Gobe, Frédéric Fourreau, Jean-Winoc Decousser

**Affiliations:** 1 Infection Control Team, Microbiology Department, University Hospital Henri Mondor, Assistance Publique—Hôpitaux de Paris, Créteil, France; 2 University Paris Est Créteil, Health Faculty, EA 7380 DYNAMYC, Créteil, France; Bingol University: Bingol Universitesi, TURKEY

## Abstract

**Background:**

During the COVID-19 pandemic, the suspension of relatives’ visits was a common measure in healthcare facilities to prevent the spread of the virus among patients. This measure caused significant adverse consequences for hospitalized patients. Volunteers’ intervention was an alternative but could also lead to cross transmission events.

**Aims:**

in order to secure their intervention with patients, we implemented an infection control training to evaluate and to improve the knowledge of volunteers about infection control measures.

**Method:**

We performed a before-after study in a group of five tertiary referral teaching hospitals in the suburbs of Paris. A total of 226 volunteers from three groups (religious representatives, civilian volunteers and users’ representatives) were included. Basic theoretical and practical knowledge about infection control, hand hygiene, and glove and mask use were evaluated just before and immediately after a three-hour training program. The contribution of the characteristics of the volunteers to the results was studied.

**Findings:**

The initial conformity rate for theoretical and practical infection control measures ranged from 53% to 68%, depending on the participants’ activity status and education level. Some critical shortcomings in hand hygiene as well as mask and glove wearing putatively endangered the patients and volunteers. Surprisingly, serious gaps were also identified among volunteers who experienced care activities. Regardless of their origin, the program significantly improved both their theoretical and practical knowledge (p<0.001). Real-life observance and long-term sustainability should be monitored.

**Conclusions:**

To become a secure alternative to relatives’ visits, volunteers’ interventions must be preceded by the assessment of their theoretical knowledge and practical skills in infection control. Additional study, including practice audit, must confirm the implementation of the acquired knowledge in the real-life.

## Introduction

The COVID-19 pandemic had tragic consequences in health care facilities (HCFs) and nursing homes; the local spread of the virus inside establishments led to a substantial number of cases and associated deaths [1, 2]. Among the infection control measures to prevent the spread of the virus, the limitation and/or suspension of relatives’ visits was implemented in numerous HCFs [3, 4]. These measures presented adverse consequences for both the physical and the mental health of the patients and the residents [5].

Alternatives to relatives’ visits exist. In addition to digitized forms (connected tablet/computer or cell phone), the intervention of volunteers could compensate at least in part for the lack of person-to-person interactions with close family. These volunteers belonged to different groups or associations and included religious representatives, civilian volunteers and user representatives.

The volunteers went from room to room and could participate in the cross-transmission of pathogens, especially during episodes of intense community spread of the virus [6]. They had to deal with the risk of cross-transmission and have the basics of microbiology, transmission pathways and infection control [6]. The lack of data regarding their initial level of knowledge and the putative contribution of a training session for all or some of them to increase their knowledge prevents the implementation of an appropriate strategy. In the following work, we implemented a dedicated intervention to secure the actions of the different categories of volunteers in a university hospital group. After an initial evaluation of their knowledge and attitudes, we measured the contribution of a quick training program leading to the dispensation of an “Infection control passport”. We identified the characteristics of the volunteers that influenced their initial level of competence and subsequent progression.

## Material methods

We performed an anonymous, multicenter prospective study based on both theoretical and practical evaluations.

### Study population

The Hôpitaux Universitaires Henri Mondor (HU-HMN) is a 2814-bed tertiary care center including five different hospitals located in the eastern suburbs of Paris. It belongs to the Assistance Publique–Hôpitaux de Paris (AP-HP) network, which is the largest hospital network in Europe and includes 39 health care facilities. Due to the SARS-CoV-2 pandemic, all physical visits to hospitalized patients were forbidden from March 2020 to July 2020 in all five hospitals. Complaints were raised immediately from patients and their families to point out their feelings of loneliness and abandonment. To alleviate this interdiction, different categories of volunteers who had visited the patients before the pandemic proposed to return to visiting patients. To secure both the volunteers and the patients, the infection control team and the service users’ executive proposed implementing an “infection control passport” for all volunteers who wanted to visit hospitalized patients. This passport was provided after a three-hour training and gave volunteers permission to visit all hospitalized patients, excluding those in quarantine for current SARS-CoV-2 infection and those from wards with running COVID-19 clusters. From August 2020, the infection control team organized several training sessions in the different hospitals of the group that included a pre- and postquestionnaire and evaluation of theoretical and practical training.

### Study design

#### Training sessions

*The pre- and postquestionnaires* were identical: assertions about microbes, microbe transmission, and infection control measures to prevent hospital-acquired infection (hand hygiene, use of personal protective equipment, etc.) were proposed, and the participants had to determine their veracity (please see the S1 File). Demographic data included the type of volunteer (religious representatives/civilian volunteers/users), sex, age, professional activity/retirement, education level (< or > 3 years after the high school diploma), history of infection control training whatever the time between this training and the current study, and history of health care practice. As a part of this study, the testing was performed just before and just after the training session. The responses to the pre- and postquestionnaires were anonymously collected and analyzed according to the demographic data.

The *theoretical training* consisted of a projected presentation about the following topics: general information on microorganisms and microbiota, the transmission pathways of microorganisms, definitions and examples of hospital-acquired infections, and fighting microorganism transmission (hand hygiene, respiratory hygiene, vaccinations and infection control measures).

The *practical training* included the use of hydroalcoholic hand rubbing and the donning and duffing of a facial mask under the supervision of an infection control team member (please see below).

#### Practical knowledge assessment

In addition to the previously reported pre- and postquestionnaires, the practical assessment included hand hygiene and facial mask manipulation as in the practical training. The main targeted points were the fulfillment of the hand hygiene prerequisites (i.e., lack of rings and bracelets, lack of gloves), the quantity of hydroalcoholic solution that was drawn (i.e., a palm), the realization of the different steps of hand rubbing according to the WHO recommendations, the minimum hand rubbing duration (i.e., 30 seconds), the use of hand sanitizer before picking a mask from the box, the use of the mask’s right side and pinching the stiff edge to the shape of the nose [7].

At the end of the training session, each participant received an “infection control passport” that gave permission to intervene successively in different patients’ rooms. No minimum score was required to obtain this passport, and each volunteer completed the training only once.

#### Statistical analysis

The primary endpoint of the analysis was the increase of theoretical and practical knowledge after the training session. From a pilot study we estimated the pre- and post-training level of knowledge. We used the standard statistical methods to calculate the sample size to identify at least a 15% difference in term of conformity with a two-sided 5% significance level and an 80% statistical power. The minimum size was estimated at 159 participants. Therefore, we included the successive groups of participants until their total number reached this value.

The statistical analysis was performed using the R package version 4.1.2 (2021-11-01). Knowledge before and after training according to the different variables was compared with the student’s t-test (α = 0.05). The knowledge between the different categories of volunteers was analyzed by two-way ANOVA comparison tests with a statistical significance fixed at p < 0.01 after verification of compliance with all conditions. Confidence intervals of 99% were provided for each studied parameter.

### Information and consent

This was an anonymous survey. All participants were informed and provided oral consent. Before the beginning of each training session, we provided the following information to each participant: “The anonymous data about your profile and about the evaluation results you obtain before and after the training session could be used to research purposes. If you not consent to this use, you can report it immediately on the questionnaire or to the trainer during the following month. Your decision has no consequence on the obtaining of your Infection Control Passport“.

## Results

The data set was available in the S1 Dataset.

### Characteristics of the studied population

From September 2020 to February 2022, the infection control team organized 25 training sessions in the different hospitals of the group. Due to the standardization of the training, the results of the different sessions were collected. During these sessions, 226 persons completed the training: 163 civilian volunteers (163/226, 72.1%), 51 religious representatives (51/226, 22.6%), and 12 user representatives (12/226, 5.3%). All the participant accepted to be included in the study. Regarding the three parts of the study (theoretical knowledge, hand-hygiene disinfection and facial mask manipulation) the number of paired responses for the pre- and post- training test reached the required minimal size every time (224, 191 and 176, respectively).

The characteristics of the volunteers are reported in Table 1. Briefly, the M/F sex ratio was 0.36, 47.8% had a high school education, and 52.0% were retired at the time of the questionnaire. Most of the volunteers did not have a history of professional activity in a health care setting and did not previously receive training in infection control (83.2% and 88.5%, respectively). Regarding the different volunteer classes, their corresponding numbers were insufficient to compare their characteristics.

**Table 1:** Characteristics of the 226 volunteers according to their origins.

	Total	Civilian volunteers (%)	Religious representatives (%)	Users’ representatives (%)
Number (%)	226 (100%)	163 (72.1)	51 (22.6)	12 (5.3)
Sex ratio (M/F)	0.36	0.32	0.55	0.20
High school education (%)	98 (47.3%)	68 (45.0)	28 (60.9)	2 (20.0)
Retired (%)	90 (52.0%)	67 (51.9)	17 (46.0)	6 (85.7)
History of professional activity in the healthcare setting (%)	38 (16.8%)	28 (17.2)	8 (13.8)	2 (16.7)
History of training in infection control (%)	26 (11.5%)	16 (9.8)	6 (11.8)	4 (33.3)

### Initial theoretical evaluation

Regarding the 15 questions, the rate of correct answers varied from 14.5% to 91.2%. From a global perspective, > 50% and > 66% correct answers were obtained for 11/15 and 7/15 assertions, respectively. The four assertions with the lowest observed correct rates were (1) “In the hospital, all patients with a contagious disease are identified and isolated”, (2) “Alcohol-based hand rubbing is more effective than simple hand washing (water + soap) on microorganisms”, (3) “Hydroalcoholic products offer a better tolerance on the hands than soaps” and (4) “The systematic use of gloves in the hospital reduces the risk of an outbreak”.

**Fig 1:**
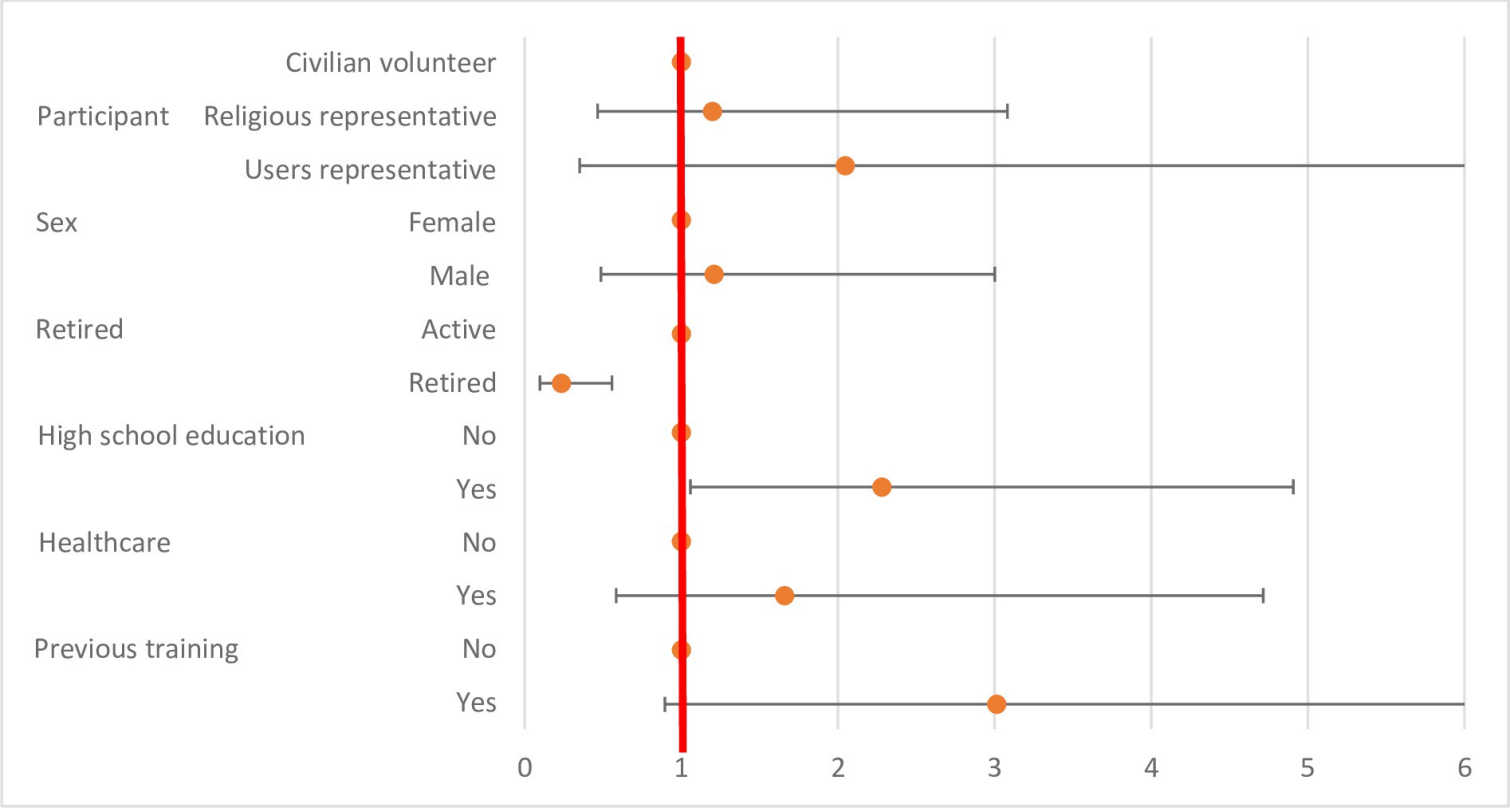
Level of knowledge before the training session according to participants’ characteristics.

The level of knowledge before the training was significantly higher according to high school education (p = 0.0057) and lower according to retired status (p<0.0001). No significant difference was identified according to the origin of the volunteers, sex, history of infection control training, or history of health care practice (p>0.01) (Table 2 and Fig 1)

**Table 2:** Level of knowledge before the training session according to participants’ characteristics.

		OR	99% confidence intervals		P value
**Categories of participants**	*Civilian volunteer*	1	**Reference**		
	*Religious representative*	1,20	0,47	3,08	0,4975
	*Users representative*	2,04	0,35	11,90	1,0551
**Sex**	*Female*	1	**Reference**		
	*Male*	1,21	0,49	3,00	0,5874
**Retired**	*Active*	1	**Reference**		
	*Retired*	0,23	0,10	0,56	**<0,0001**
**High school education**	*No*	1	**Reference**		
	*Yes*	2,28	1,06	4,91	**0,0057**
**History of professional activity in the healthcare setting**	*No*	1	**Reference**		
	*Yes*	1,66	0,58	4,72	0,2087
**History of previous training in infection control**	*No*	1	**Reference**		
	*Yes*	3,01	0,90	10,14	0,0190

### Evolution of the theoretical evaluation after the training program

**Fig 2:**
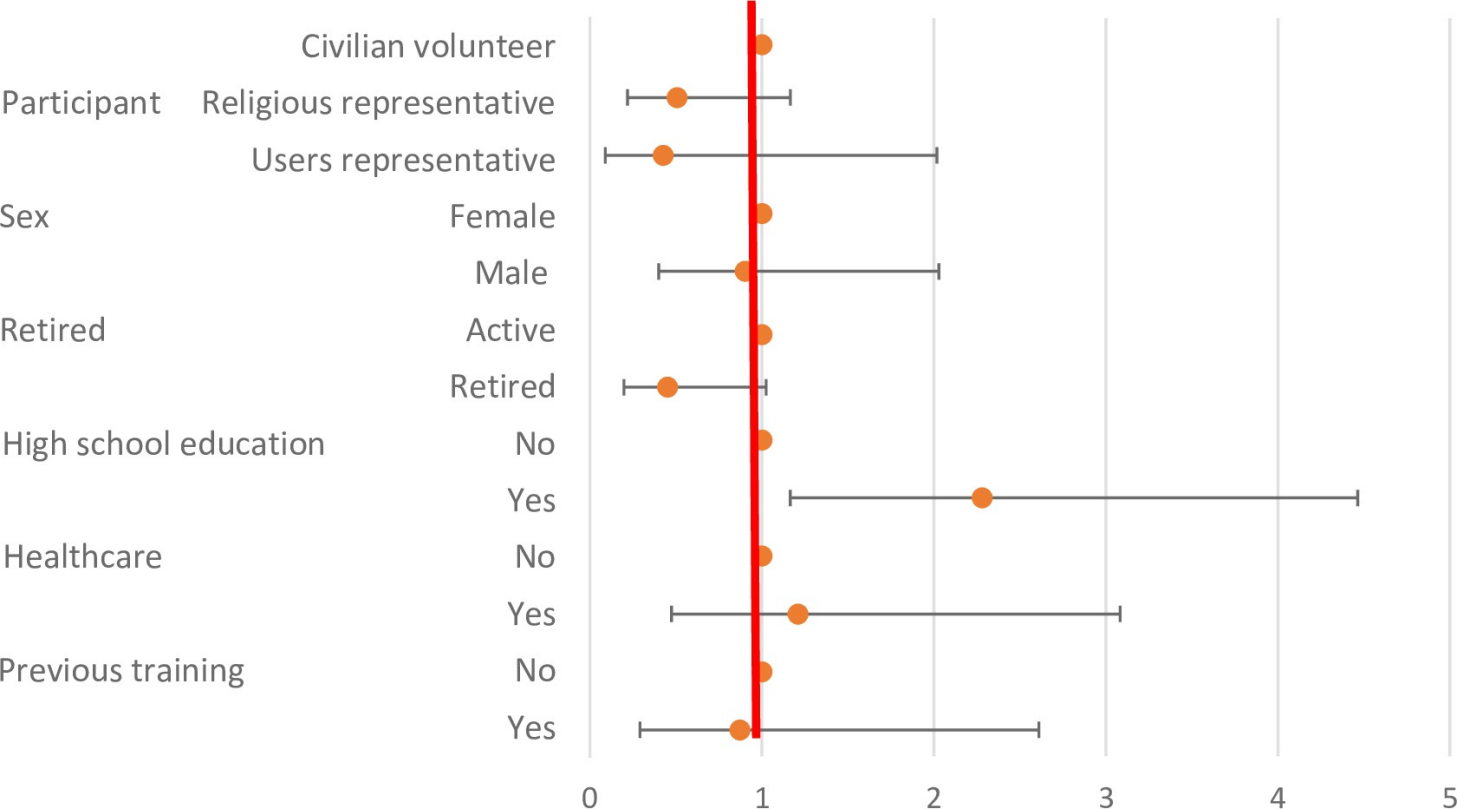
Level of knowledge after the training session according to participants’ characteristics.

The level of knowledge after the training was significantly higher according to high school education (p = 0.0016). No significant differences were identified according to the origin of the volunteers, sex, the retired status history of infection control training, or history of health care practice (p>0.01) (Table 3 and Fig 2).

**Table 3:** Level of knowledge after the training session according to participants’ characteristics.

		OR	99% confidence intervals		P value
**Categories of participants**	*Civilian volunteer*	1	**Reference**		
	*Religious representative*	0,505	0,219	1,165	0,0349
	*Users representative*	0,425	0,090	2,018	0,1552
**Sex**	Female	1	**Reference**		
	Male	0,901	0,400	2,029	0,7390
**Retired**	Active	1	**Reference**		
	Retired	0,450	0,198	1,025	0,0124
**High school education**	No	1	**Reference**		
	Yes	2,281	1,165	4,465	**0,0016**
**History of professional activity in the healthcare setting**	No	1	**Reference**		
	Yes	1,210	0,475	3,083	0,5974
**History of previous training in infection control**	No	1	**Reference**		
	Yes	0,872	0,291	2,611	0,7456

**Fig 3:**
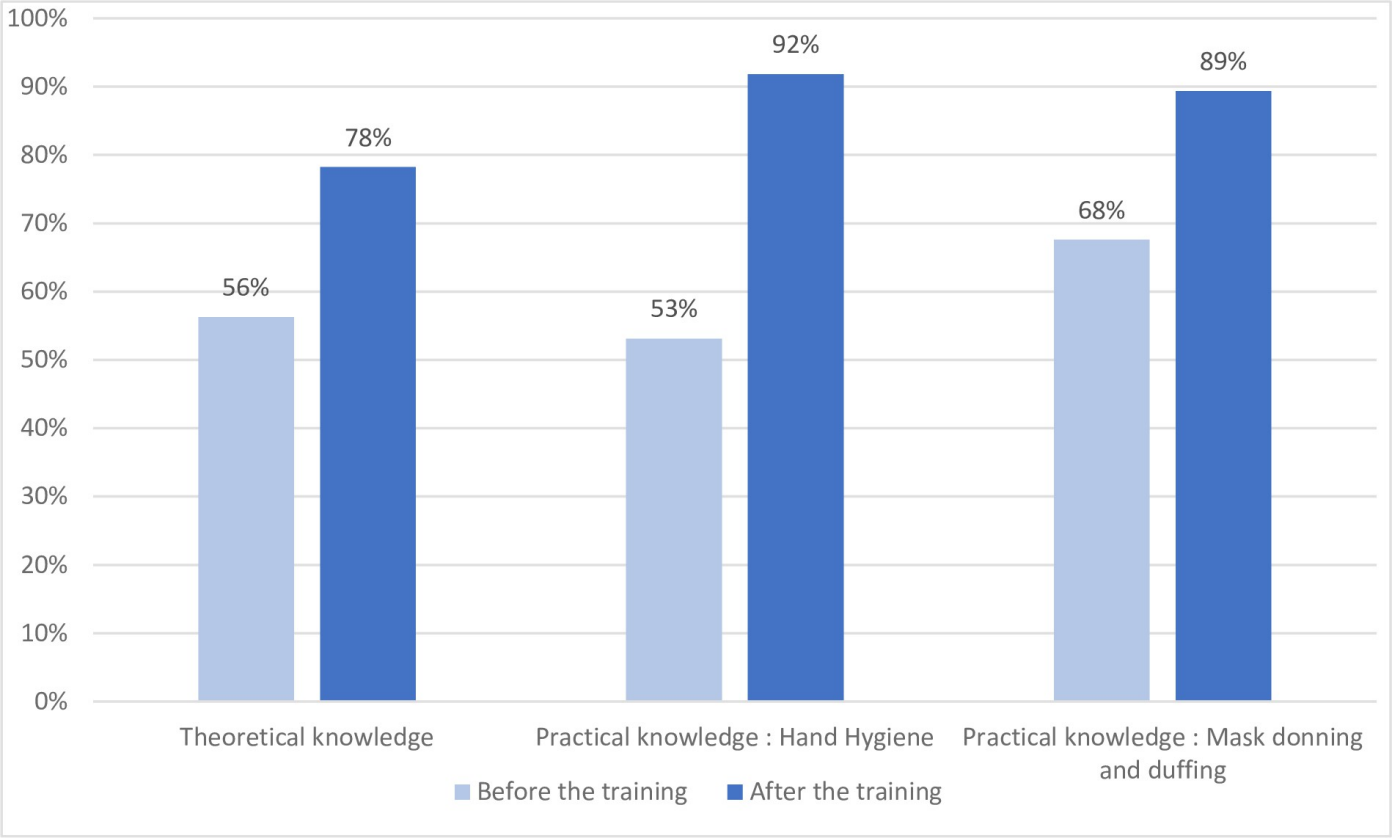
Evolution of the theoretical and practical conformity of the volunteers (N = 226) before and after the training sessions. Each improvement in theoretical and practical knowledge was significant (p <0.001).

After the training program, the rate of correct answers increased from + 5.9% to + 73.8%: The average of the differences was +22% [18.93; 25.08] (p<0.001) (Fig 3). Regarding the four assertions reporting the lowest correct answer initial rate, 3 out of 4 increased beyond 88% conformity. The high school education increased significantly the rate of correct answered (p = 0.00164) but not the other characteristics (i.e., sex, retirement status, former occupational activities in health care settings, previous infection control training or category of volunteers).

### Initial practical evaluation

#### Hand hygiene

One of the participants was brought out with care gloves to the training.

Regarding the hand rubbing technique, 5 out of 11 items did not reach a > 50% conformity rate, and 8 out of 11 did not reach a > 66% conformity rate. The three most often inaccurate items were (1) hand nails by rotating in the palm contra-lateral and vice versa, (2) rotational rubbing of left thumb clasped in right palm and vice versa and (3) backs of fingers to opposing palms with fingers interlocked (25.7%, 31.0% and 31.5% conformity, respectively). Hand rubbing lasted at least 30 s in less than 50% of the participants (48.1%).

**Fig 4:**
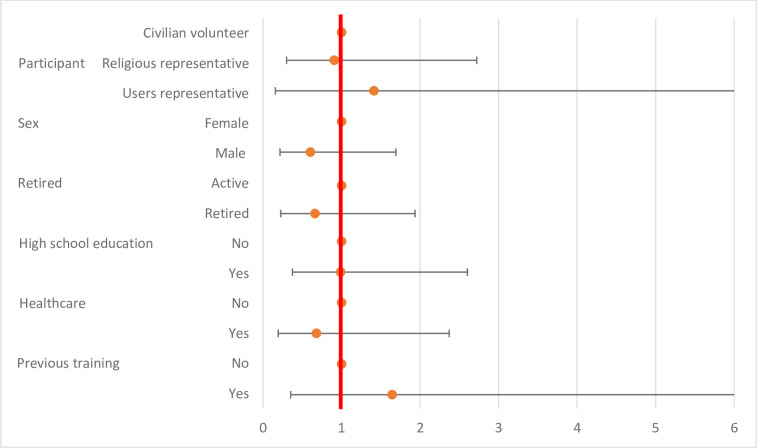
Level of conformity in Hand hygiene before the training session according to participants’ characteristics.

None of the characteristics of the participants was significantly associated to the conformity of hand hygiene (Table 4 and Fig 4).

**Table 4:** Level of conformity in Hand hygiene before the training session according to participants’ characteristics.

		OR	99% confidence intervals		P value
**Categories of participants**	***Civilian volunteer***	1	**Reference**		
	***Religious representative***	0,90	0,30	2,72	0,8133
	***Users representative***	1,41	0,16	12,69	0,6837
**Sex**	Female	1	**Reference**		
	Male	0,60	0,22	1,69	0,2042
**Retired**	Active	1	**Reference**		
	Retired	0,66	0,23	1,94	0,3166
**High school education**	No	1	**Reference**		
	Yes	0,99	0,37	2,60	0,9730
**History of professional activity in the healthcare setting**	No	1	**Reference**		
	Yes	0,68	0,19	2,37	0,4217
**History of previous training in infection control**	No	1	**Reference**		
	Yes	1,64	0,35	7,65	0,4017

#### Mask donning and duffing

**Fig 5:**
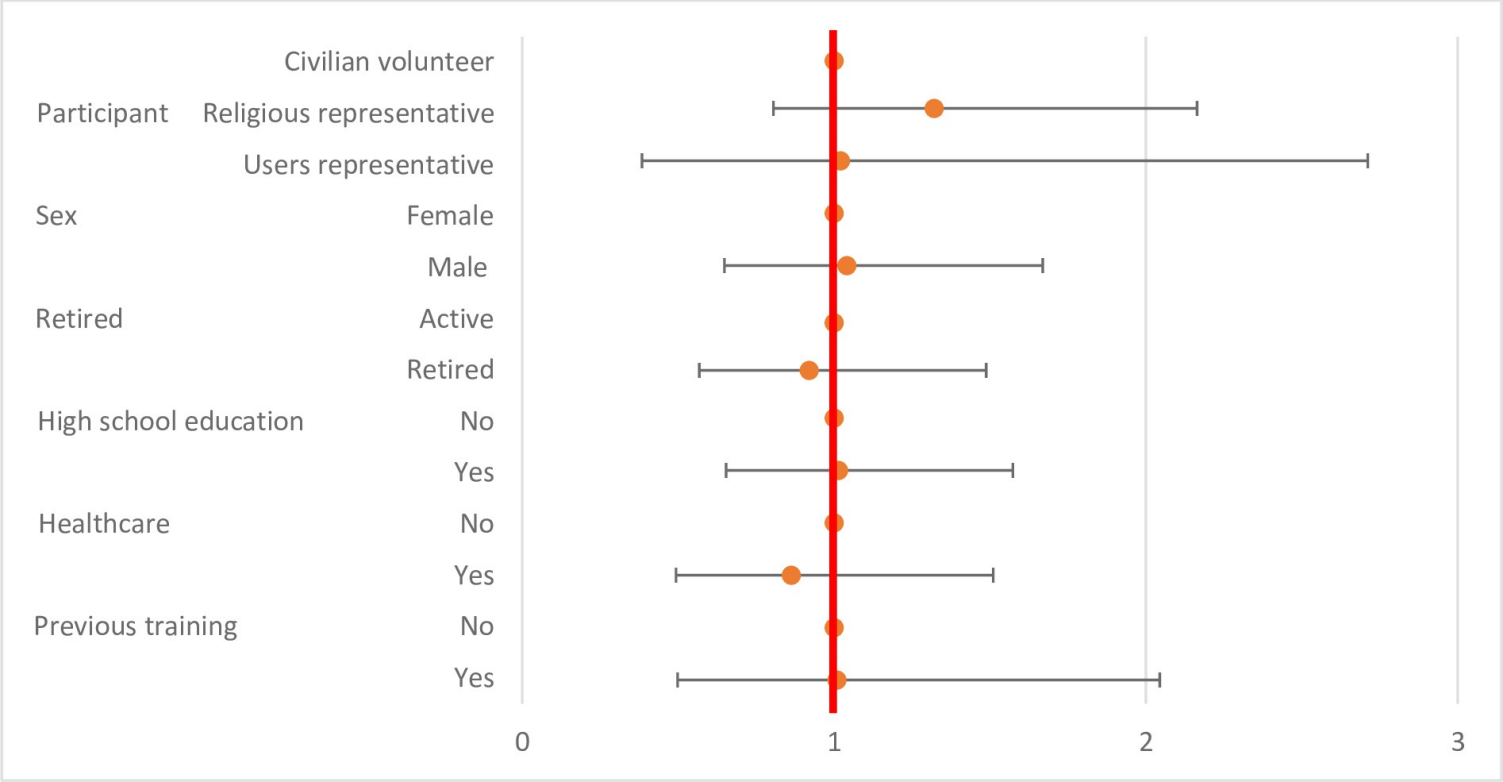
Level of conformity in mask donning and duffing before the training session according to participants’ characteristics.

Before the training, the lowest average compliance percentages were obtained for the following items: “to perform alcohol-based hand rubbing before duffing a mask” (33.3% conformity) and “to perform alcohol-based hand rubbing before picking a mask from the box” (48.4% conformity). None of the characteristics of the participants was significantly associated to the initial proficiency level of surgical mask handling (Table 5 and Fig 5).

**Table 5:** Level of conformity in mask donning and duffing before the training session according to participants’ characteristics.

		OR	99% confidence intervals		P value
**Categories of participants**	*Civilian volunteer*	1	**Reference**		
	*Religious representative*	1,32	0,81	2,16	0,1449
	*Users representative*	1,02	0,38	2,71	0,9568
**Sex**	Female	1	**Reference**		
	Male	1,04	0,65	1,67	0,8273
**Retired**	Active	1	**Reference**		
	Retired	0,92	0,57	1,49	0,6485
**High school education**	No	1	**Reference**		
	Yes	1,01	0,65	1,57	0,9323
**History of professional activity in the healthcare setting**	No	1	**Reference**		
	Yes	0,86	0,49	1,51	0,4945
**History of previous training in infection control**	No	1	**Reference**		
	Yes	1,01	0,50	2,04	0,9725

### Evolution of the practical evaluation after the training program

#### Hand hygiene

**Fig 6:**
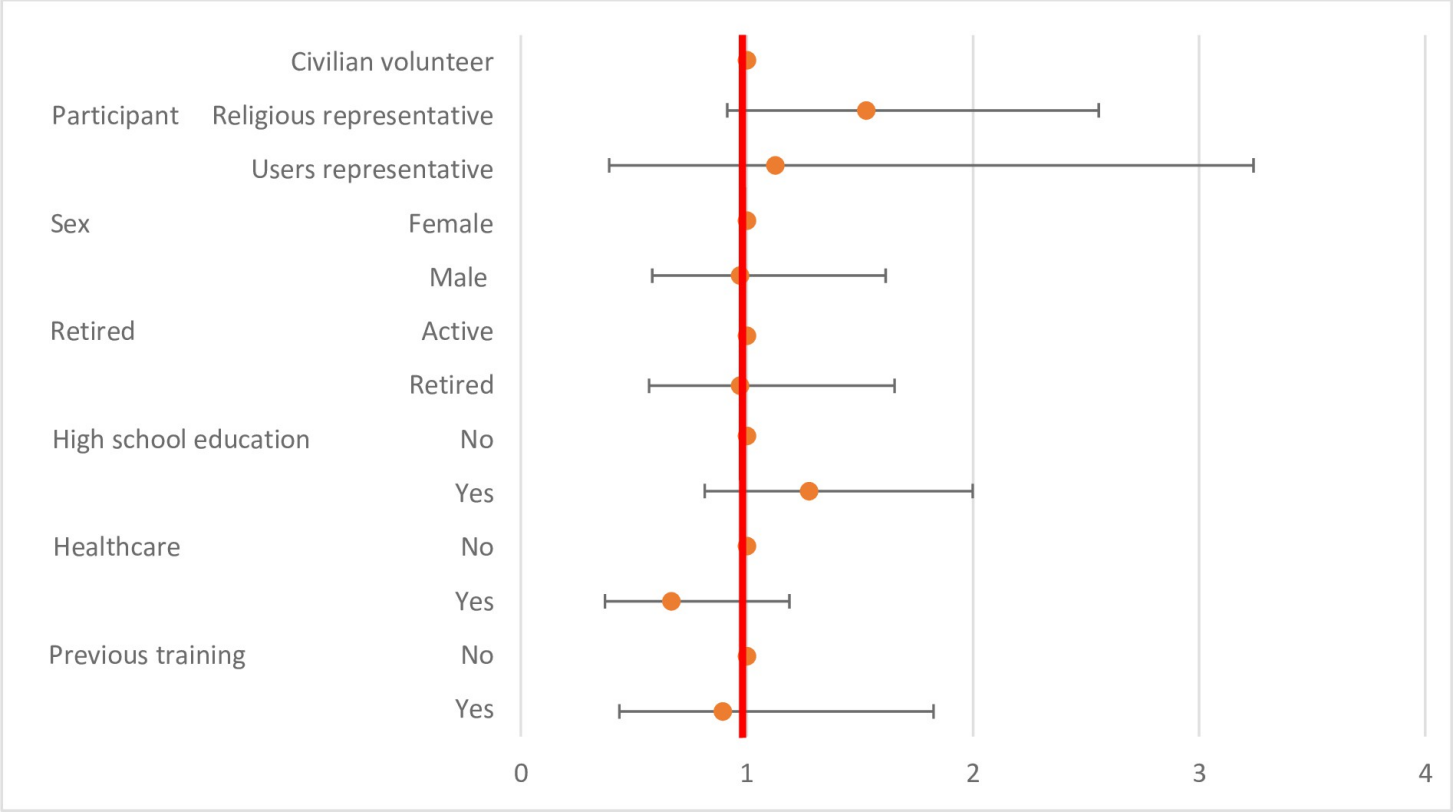
Level of conformity in Hand hygiene after the training session according to participants’ characteristics.

None of the characteristics of the participants was significantly associated to the conformity of hand hygiene after the training program (Table 6 and Fig 6).

**Table 6:** Level of conformity in Hand hygiene after the training session according to participants’ characteristics.

		OR	99% confidence intervals		P value
**Categories of participants**	*Civilian volunteer*	1	**Reference**		
	*Religious representative*	1,53	0,91	2,56	0,0334
	*Users representative*	1,13	0,39	3,24	0,7713
**Sex**	Female	1	**Reference**		
	Male	0,97	0,58	1,61	0,8715
**Retired**	Active	1	**Reference**		
	Retired	0,97	0,57	1,65	0,8765
**High school education**	No	1	**Reference**		
	Yes	1,28	0,81	2,00	0,1607
**History of professional activity in the healthcare setting**	No	1	**Reference**		
	Yes	0,66	0,37	1,19	0,0688
**History of previous training in infection control**	No	1	**Reference**		
	Yes	0,89	0,44	1,83	0,6785

The conformity rate increased dramatically after the training program for all 11 items (from + 5.9% to +57.9%), with all but one item exceeding 80%. The average of the differences was +38.2% [33.62; 42.8]; this increase was significant (p<0.001) (Fig 3). The worst item was “rotational rubbing, backward and forward with clasped fingers of right hand in left palm and vice versa”, which increased from 25.7% to 76.5%. The duration of hand rubbing lasted 30 s or more for 95.9% of the participants. None of the characteristics of the participants were associated with a statistically significant difference after the training program.

#### Mask donning and duffing

**Fig 7:**
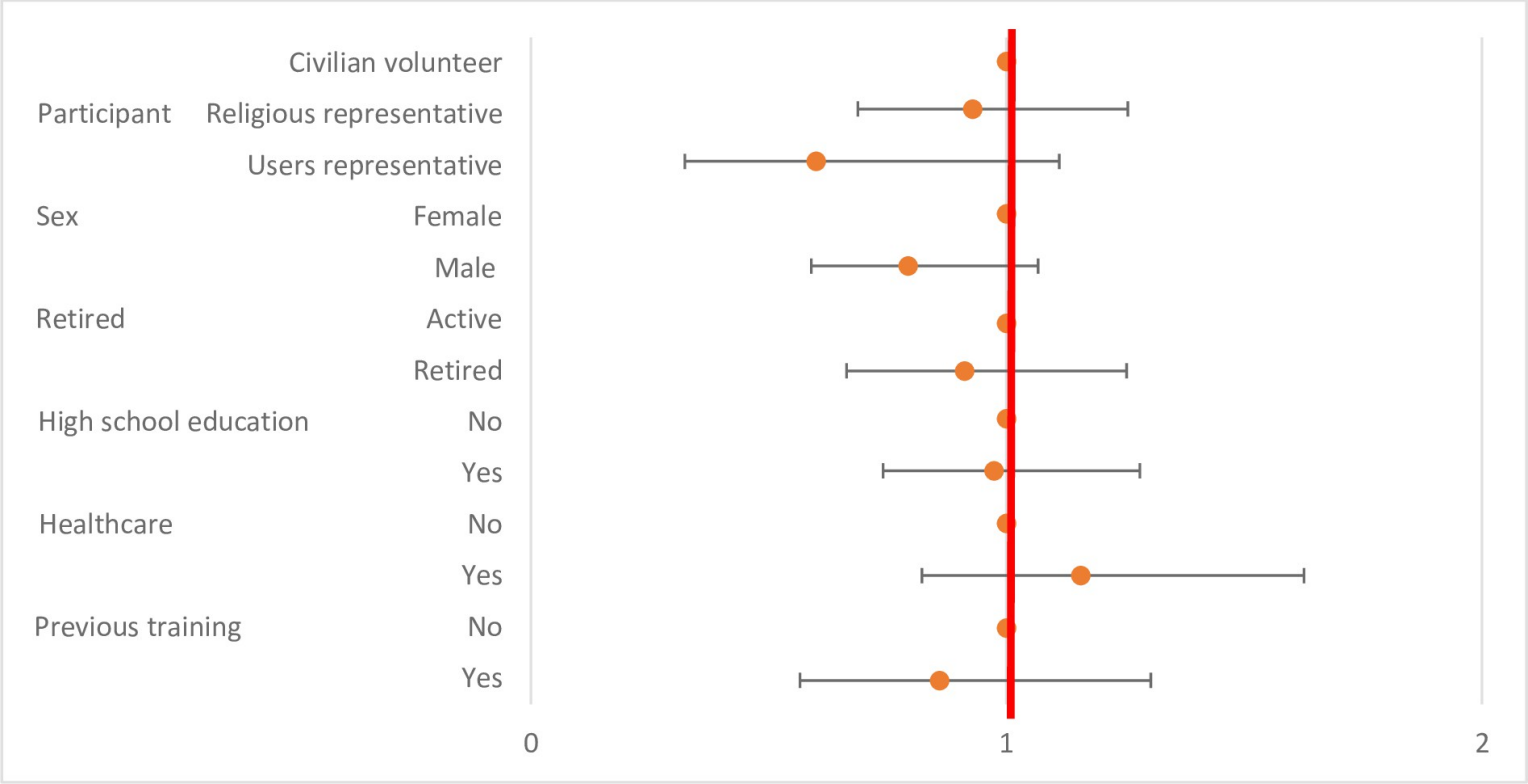
Level of conformity in mask donning and duffing after the training session according to participants’ characteristics.

None of the characteristics of the participants was significantly associated to the proficiency level of surgical mask handling after the training session (Table 7 and Fig 7).

**Table 7:** Level of conformity in mask donning and duffing after the training session according to participants’ characteristics.

		OR	99% confidence intervals		P value
**Categories of participants**	*Civilian volunteer*	1	**Reference**		
	*Religious representative*	0,93	0,69	1,26	0,5253
	*Users representative*	0,60	0,32	1,11	0,0323
**Sex**	Female	1	**Reference**		
	Male	0,79	0,59	1,07	0,0431
**Retired**	Active	1	**Reference**		
	Retired	0,91	0,66	1,25	0,4500
**High school education**	No	1	**Reference**		
	Yes	0,97	0,74	1,28	0,8024
**History of professional activity in the healthcare setting**	No	1	**Reference**		
	Yes	1,16	0,82	1,63	0,2691
**History of previous training in infection control**	No	1	**Reference**		
	Yes	0,86	0,57	1,30	0,3442

The conformity rate increased after the program training for all 5 items (from + 7.6% to +41.7%), with all but one exceeding 75%. The average of the differences was + 21.14% [16.78; 25.49]; this increase was significant (p<0.001) (Fig 3).

## Discussion

The control of microorganism transmission inside health care facilities is a daily challenge for infection preventionists. Viruses pose a particularly serious threat because of their intensive community circulation and the varying ways of transmission (directly through saliva droplets or indirectly through hands or fomites). SARS-CoV-2, flu, RSV, and norovirus are the leading causes of viral outbreaks. In addition to the struggle against their transmission within the premises, limiting the introduction of viruses from the community inside HCFs is a relevant action. During the SARS-CoV-2 pandemic, local and national guidelines produced a drastic reduction in visits, with some facilities banning them [1–4]. Visitors could be a reservoir of virus, especially if they are asymptomatic shedders; Paasarelli et al. reported two contaminations of inpatients by asymptomatic visitors despite a policy of universal masking [1, 2–8]. Although the contribution of visitor restrictions to the COVID-19 pandemic has been discussed, the observance of infection control measures (social distancing, mask wearing, hand hygiene) may be imperfect during visits: in the study of Wee et al., the implementation of visitors restrictions was associated to a significant decrease of the cumulative incidence of hospital-associated respiratory viral infections from 9.69 to 0.66 case per 10,000 patient-days [9]. A strategy based on screening (PCR or antigen detection) before a visit is costly and time-consuming if health care givers must check the results [10]. oreover, this policy is putatively conflicting. The consequences of this limitation were rapidly highlighted: patients and residents suffered from loneliness and depression, leading to the risk of a shift toward a failure to thrive and its consequences in terms of morbidity and mortality [5, 11, 12]. Keeping hospitalized people in touch with their social environment could be promoted through digitalized interactions or the strict implementation of infection control measures for physical interactions, including the promotion of regular screening and immunization of relatives [10]. An alternative was interventions by volunteers from different origins who could compensate for the absence or remoteness of relatives. These volunteers may interact with different patients from different wards, unlike the relatives of a hospitalized patient [6]. Therefore, the risk of cross-transmission added to the risk of the importation of pathogens from the community [6]. Considering volunteers as long-term partners, we implemented practical and theoretical training to evaluate and improve knowledge and adequate behaviors in terms of infection control. We formalized participation in this traineeship to obtain an “infection control passport”. To the best of our knowledge, our approach is new and, to date, unique. Considering that dealing with the associated risk of volunteer intervention is a matter for infection control teams, this training was mandatory; no new volunteers were allowed to visit patients before obtaining this passport. Several training sessions were implemented at regular intervals in the five different hospitals. The training program was developed by the central infection control team and performed in a uniform way in time and space.

Regarding the initial level of competency, some volunteers had serious shortcomings in knowledge and practices; it was not possible to identify a particular category of volunteers that absolute required or could be exempt from the training. This finding is important to implement a cost-effective strategy. For example, the misuse of gloves is a leading cause of cross-transmission: gloves cannot be sanitized between patients, and their use must be limited to blood/body fluids/mucosal or injured skin contacts [13]. However, two-thirds of the volunteers believed that the systematic use of gloves in the hospital reduced the risk of an outbreak. Surprisingly, one volunteer attended the training wearing care gloves. In addition, a large majority of trainees did not support the use of alcohol-based hand rub solutions as a first-line strategy for hand hygiene and favored the use of soap and water. It is well established that the antibacterial efficiency and tolerance of hand washing are inferior to hydroalcoholic friction: in term of efficiency, the switch from soap and water to alcohol-based hand rib reduce hospital associated infection rates by 50% [14]. For the majority of the participants, the wearing of a wedding ring or the presence of visible soil on the hands did not reduce the efficacy of alcohol-based hand rub solutions. Some aspects of hand hygiene were poorly controlled, leading to partially effective hand disinfection. These gaps in the basis of infection control (i.e., hand hygiene) are quite worrisome for people who come and go between vulnerable and colonized patients. Regarding mask wearing, similar shortcomings were identified that could lead to contamination of the patient and/or the volunteer. For example, less than 50% of the volunteers performed hand hygiene before picking a mask from the box, which could result in contamination, as proven for disposable gloves: indeed, in the study of Assadian et al. the mean colony-forming unit count present on gloves reached 688 at 6 weeks after opening the box [15].

These gaps are particularly worrisome. The training sessions began in September 2020, 6 months after the beginning of the SARS-CoV-2 pandemic. In France, as in numerous other countries, the Health Ministry implemented widespread information campaigns through various media (TV, social media, etc.) to promote hand hygiene. Our study noted the necessity of dedicated training for volunteers. As expected, educational level contributed to performance before and after the training session. Similar improvement was observed for patient education: in the recent review of Hammoud et al., an improvement of 48.1% of hand hygiene knowledge was reported among patients after an education program [16] Retired people exhibited a lower level of knowledge before the training; this could be explained by their withdrawal from workforce, a disconnection from the real life or by their excessive approach of infection control measures due to their own susceptibility to COVID-19. Nevertheless, the training session bridged the differences and allowed us to increase the performance of all the attendees regardless of their characteristics. In our institution, visits are still restricted in case of respiratory viral infection in a patient or when a cluster occurs. The next step is to allow well-trained volunteers to visit in these risky situations.

Our study has several limitations. First, we did not implement a cutoff level, and we gave the passport to all attendees. Even though some theoretical knowledge could be improved for several attendees (e.g., about the isolation policy in hospitals), the mastery of hand hygiene and mask wearing was excellent after the training session. Thereafter, there are several pending issues that we must address. How will these infection control measures be observed in real life? The second testing was performed just after the training session; moreover, the infection control passport has no validity limit. Then will the capacities be maintained over time? An observational study based on audits should be implemented at different times after the training course. Moreover, the impact of this action on the occurrence of hospital-acquired infections and outbreaks (e.g., COVID-19 cases) was not established, and additional studies are needed. For legal reasons, we strongly promoted immunization against flu among the volunteers but did not make it mandatory. However, regarding COVID-19 legislation, all people entering an HCF had to be immunized or have had a negative test in the last 24 h.

Finally, our study was limited to volunteers involved in HCFs. We support the same approach for nursing homes and other closed facilities with groups of vulnerable people [17].

## Conclusion

The intervention of volunteers in HCFs is of interest to minimize the loneliness of patients. Nevertheless, volunteers presented several shortcomings in their knowledge and capacity to prevent cross-transmission, including those who previously worked in a health care occupation. These gaps putatively endanger the patients and the volunteers. A quick theoretical and practical training course allowed us to significantly increase the safety of visits regardless of the characteristics of the attending population. The second testing occurring just immediately after the training session, additional monitoring of their observance over time of the infection control measures must be implemented. The real impact of this program on hospital-acquired infection occurrence must be studied.

## Supporting information

S1 File Quiz and items checked during theoretical and practical evaluation. (DOCX)

S1 Dataset Minimal data set. (XLSX)
