# Supplementary material for: Volunteers, religious communities and users representatives as an alternative for visiting hospitalized patients: The importance of an infection control training
Source: PLoS One. 2023 May 22;18(5):e0286002. doi: 10.1371/journal.pone.0286002 (PMC10202297; doi:10.1371/journal.pone.0286002)
Supplement: S1 File — (DOCX) [file pone.0286002.s001.docx]

***Supplementary materials***

**SD 1: Quiz and Items checked during theoretical and practical evaluation**

**Quiz about microorganisms and transmission of infections in hospitals**

1. At the hospital, all patients with a contagious disease are identified and isolated. (False)

2. The bacterial flora of an inpatient is similar to that of a person outside the hospital. (False)

3. When you are sick, you are never contagious before you have clinical signs. (False)

4. In the hospital, germs are transmitted mainly by the respiratory route (droplets of saliva). (False)

5. I am not afraid of catching the patient's germs if I do not touch him and sit on his bed. (False)

6. Some germs can survive for several weeks on contaminated material or environment. (True)

7. The flu caused more deaths in 2019 than road accidents. (True)

8. At least 4 000 people die each year in France from nosocomial infection. (True)

**Quiz about the prevention of infection in hospitals**

9. I can come to the hospital even if I have respiratory symptoms since I wear a mask. (False)

10. Hydroalcoholic friction is more effective than simple hand washing (soap + water) on microorganisms. (True)

11. Hydroalcoholic hand friction should be preferred in the presence of dirt on the hands. (False)

12. Wearing an appliance does not diminish the effectiveness of hydroalcoholic hand friction. (False)

13. Hydroalcoholic products offer better tolerance on the hands than soaps. (True)

14. The systematic wearing of gloves in the hospital limits the risk of an outbreak. (False)

15. There is no need for a flu shot since all patients in the hospital are vaccinated. (False)

**Items checked during the hand hygiene practical evaluation**

1. Take a sufficient volume of hydroalcoholic solution for 30 seconds of hand rubbing

Perform the following gestures:

2. Rub hands palm to palm

3. Right palm over left dorsum with interlaced fingers and vice versa

4. Palm to palm with fingers interlaced

5. Backs of fingers to opposing palms with fingers interlocked

6. Rotational rubbing of the left thumb clasped in the right palm and vice versa

7. Hand nails by rotating in the palm contra-lateral and vice versa

8. Rotational rubbing of the wrists

9. Carry out the gesture until evaporation

10. Do not dry hands by shaking

11. Hand rubbing lasts at least 30 seconds

**Items checked during mask donning/duffing practical evaluation**

1. Hand hygiene performed before removing the mask

2. Removal of the mask from the ears

3. Hand hygiene performed before taking the mask from the box

4. Pull the mask down to cover the entire face

5. Fit the mask on the nose with the metal bar

[
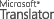
](http://www.bing.com/translator)
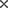


**Langue source**

Fonction : ⃝ Bénévole ⃝ Cultes ⃝ Représentant des usagers ⃝ Autre : ………………………………………
